# The Brain‐Gut Axis in Parkinson's Disease Pathology

**DOI:** 10.1002/cph4.70137

**Published:** 2026-03-29

**Authors:** Kudret Selin Ozkaya, Kirsteen N. Browning

**Affiliations:** ^1^ Department of Neuroscience and Experimental Therapeutics Penn State University—College of Medicine Hershey Pennsylvania USA

## Abstract

Parkinson's disease, characterized by the motor deficits that result from the loss of dopaminergic neurons in the Substantia Nigra pars compacta, is the second most common neurodegenerative disorder worldwide. Parkinson's disease is also commonly associated with severe non‐motor symptoms, including hyposmia and sleep disorders, as well as gastrointestinal dysfunction and dysregulation of the brain‐gut axis. Increasing evidence indicates that pathology in the “body‐first” subtype of Parkinson's disease may originate in the gastrointestinal (GI) tract and then spread to the brain via the vagus nerve. GI dysfunction may also arise, however, from “top‐down” or “brain‐first” mechanisms, reflecting bidirectional brain‐gut interactions. Systemic environmental factors, such as exposure to pesticides and heavy metals, are hypothesized to initiate this pathology and promote α‐synuclein (α‐syn) misfolding and the formation of Lewy Bodies. A growing body of evidence suggests, however, that oxidative stress and glial activation may emerge in the enteric nervous system and the dorsal motor nucleus of the vagus long before the onset of neurodegeneration, and that these early changes may be sufficient to drive the retrograde spread of pathology to higher brain regions. The purpose of this review is to discuss the progression of PD pathology across the brain‐gut axis, providing insights into the contribution of oxidative stress and glial activation to neuronal loss, and highlighting the importance of these mechanisms for potential therapeutic intervention at the earliest stages of PD.

## Graphical abstract

Exposure to environmental toxins induces gut dysbiosis, increases intestinal epithelial barrier permeability, immune activation, and oxidative stress, promoting α‐syn misfolding and propagation. Brainstem DMV neurons exhibit α‐syn pathology, dysregulating neuronal excitability and vagal control of GI functions. Dopaminergic SNpc neurons exhibit α‐syn pathology, increased oxidative stress, glial activation, mitochondrial dysfunction, and neurodegeneration. Figure legend: Representative schematic illustrating route of pathology propagation and anatomical connectivity between the GI tract, brainstem (via the vagus nerve) and—the SNpc (via the nigro‐vagal pathway). Vagal afferent fibers relay sensory information from the GI tract to the NTS, which assimilates this information with inputs from brainstem, midbrain, and higher brain regions before relaying the integrated signal to the adjacent DMV. Preganglionic parasympathetic neurons of the DMV innervate postganglionic neurons within the ENS to regulate GI functions. The SNpc modulates DMV neuronal activity, hence parasympathetic output, via dopaminergic inputs to activate D1‐like receptors. The right panels indicate region‐specific pathological changes observed in PD. In the SNpc, (top panel), dopaminergic neurons exhibit α‐syn pathology, increased oxidative stress, glial activation, mitochondrial dysfunction and neurodegeneration, and subsequent loss of neuromelanin. In the DMV, (middle panel) cholinergic neurons exhibit α‐syn pathology, increased oxidative stress, glial activation, mitochondrial dysfunction, leading to increased neuronal excitability and disrupted GI functions (i.e., delayed gastric emptying, altered GI motility). In the ENS, (bottom panel) exposure to environmental toxins and altered gut microbiota increase epithelial barrier permeability, immune activation and oxidative stress, which promotes α‐syn misfolding and propagation. Image created with Biorender under license (KNB).

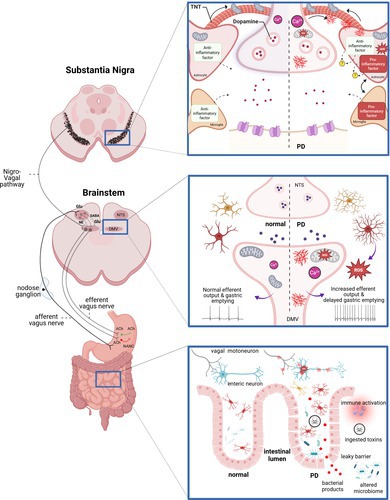

## 1 Introduction

Parkinson's Disease (PD) is the second most common neurodegenerative disorder, currently affecting nearly 10 million people worldwide, with the number of patients affected estimated to double by 2040, making PD the neurodegenerative disease with the fastest‐growing prevalence (Su et al. 2025). Classically, PD is characterized by motor deficits such as bradykinesia, tremors, and rigidity, which result from the loss of dopaminergic neurons in the substantia nigra pars compacta (SNpc) (Heng et al. 2023). Accumulating evidence suggests that PD may progress along two distinct pathogenic pathways; in the “body‐first” subtype, pathology originates in the gastrointestinal (GI) tract and subsequently spreads, likely via the vagus nerve, into the brain, in contrast to the “brain‐first” subtype where the pathology begins in the brain itself. The brain‐first etiology is more commonly associated with monogenic causes and accounts for approximately 5%–10% of PD cases, while the remainder represents the idiopathic body‐first etiology, which is strongly linked to environmental factors including exposures to herbicides/pesticides, heavy metals, and drinking of well‐water. Emerging evidence also suggests that microplastics as well as per‐ and poly‐fluoroalkyl substances (PFAS; ‘forever chemicals’) can also contribute to the etiology of PD by inducing oxidative stress, mitochondrial dysfunction, and neuroinflammation (Prüst et al. 2020) as well as aggregation of misfolded α‐syn and to dopaminergic neuron loss (Liu et al. 2023). Accordingly, approximately 80% of all PD patients experience non‐motor symptoms, including hyposmia, orthostatic hypotension, rapid eye movement sleep behavioral disorders, cognitive deficits, and GI dysfunction (Poewe 2008; Travagli et al. 2020). Not only do these non‐motor symptoms decrease patient quality of life, but these symptoms are rarely targeted by the current standard of care which is primarily focused on dopamine replacement therapy. A growing body of evidence suggests that non‐motor symptoms, particularly GI disturbances, often precede motor symptoms and can therefore represent the earliest pathological changes in disease progression (Gelb et al. 1999; Cersosimo et al. 2012).

Although GI dysfunctions in PD patients were first noted by Dr. James Parkinson in 1817—who stated that “the bowels, which had been all along torpid, now, in most cases, demand stimulating medicines of very considerable power…” − it was not until 2003 that an anatomical staging model was described in which disease pathology was suggested to initiate outside the brain (Parkinson 2002). Evidence from both clinical and preclinical studies indicates that pathological changes in PD can emerge in peripheral tissues before central neurodegeneration, consistent with the prodromal appearance of non‐motor, including GI, symptoms. Accordingly, this review will focus on pathological processes contributing to the body‐first model of disease progression, emphasizing changes along the brain‐gut axis.

## 2 Parkinson's Disease

Pathologically, PD is characterized by Lewy bodies and Lewy neurites, which are composed of misfolded α‐syn protein. Although the physiological role of α‐syn remains to be elucidated, studies suggest a potential role in synaptic vesicle trafficking and neurotransmitter release, given its localization to presynaptic terminals (Iwai et al. 1995). Synucleins were identified initially by their reactivity on immunoblots of human brain extracts using an antibody that recognizes tau protein, leading to the discovery of two distinct synuclein proteins containing 140 and 134 amino acids in length, later termed β‐synuclein and α‐synuclein, respectively (Jakes et al. 1994). Shortly thereafter, genome scans conducted in a large Italian cohort identified mutations on chromosome 4q21‐q23 of the SNCA gene which encodes α‐syn, marking this as the first genetic evidence implicating α‐syn in PD etiology (Polymeropoulos et al. 1996). Subsequent analyses of postmortem PD tissue sections identified α‐syn as the primary component of Lewy bodies and demonstrated that misfolded α‐syn forms the fibrils in Lewy bodies (LB) and Lewy neurites, prompting the adoption of α‐syn‐specific antibodies, rather than ubiquitin, for neuropathological detection of PD pathology (Wakabayashi et al. 2000).

Despite the central role of α‐syn in PD pathology, the mechanism behind its misfolding and aggregation, especially in sporadic PD, remains incompletely understood. Although the exact cause of PD may be unknown, excess production of reactive oxygen species (ROS) has been linked strongly to protein misfolding, α‐syn aggregation, mitochondrial dysfunction, and inflammatory responses leading to cellular dysfunction, neuronal vulnerability and neurodegeneration, which are observed in multiple regions affected by PD (Sies 2020). While α‐syn was established as the pathological hallmark of PD, the question remains as to where—and how—the pathology originates. Familial studies have suggested that hereditary causes of PD are rare, with studies of monozygotic and dizygotic twin brothers reporting that genetic factors do not play a significant causal role in PD, particularly for those with disease onset after the age of 50 (Tanner et al. 1999). This prompted closer investigation into those factors underlying α‐syn misfolding in the more common sporadic cases, with environmental exposure(s) having been proposed as contributors to the etiology of sporadic PD (Uitti et al. 1993).

Early neuropathological studies reported that nigral damage (i.e., dopaminergic neuronal loss in the SNpc) is often accompanied by extranigral pathology and that pathophysiology follows a topographical pattern (Braak et al. 1994, 1996; Braak and Braak 2000). Braak and colleagues proposed a staging hypothesis in which an ingested “unknown pathogen” enters the enteric nervous system (ENS), leading to the misfolding and retrograde transportation of α‐syn to the central nervous system (CNS) via the vagus nerve (Braak et al. 1994, 1996; Braak and Braak 2000). The dorsal motor nucleus of the vagus (DMV), the brainstem nucleus which provides the preganglionic parasympathetic extrinsic innervation to the GI tract and is responsible for regulation and modulation of several GI functions including gastric emptying, motility, and intestinal transit (Browning and Travagli 2019; Travagli and Anselmi 2016), serves as the first source of entry of misfolded α‐syn into the CNS, from which it progresses to the SNpc via the monosynaptic nigro‐vagal pathway, and eventually to higher brain region (Anselmi et al. 2017). Support for the idea of transneuronal prion‐like transport of pathology also came from postmortem analyses of PD patients who received nigral grafts, where LB pathology was seen in the transplanted neurons years after the implantation (Li et al. 2008; Kordower et al. 2008). This model suggests that PD pathology follows a temporal sequence that aligns with the progression of both motor and non‐motor symptoms.

Despite support from clinical and preclinical studies, Braak's hypothesis has also been criticized because some patients exhibit α‐syn pathology in a sequence different to that proposed by the staging hypothesis, and SNpc degeneration has been shown to appear before brainstem or ENS pathology (Jellinger 2009; Burke et al. 2008; Kalaitzakis et al. 2008; Annerino et al. 2012). Additional concerns exist regarding the selectivity and specificity of the immunohistochemical methods used to detect misfolded α‐syn, as well as the determination of stages by the presence of Lewy pathology, implying that the Braak staging may reflect patterns of selective neuronal susceptibility rather than a strict sequential spread of pathology (Jellinger 2009; Braak et al. 2003).

Nevertheless, Braak's hypothesis implies that both brain‐first and body‐first pathologies occur and contribute to the heterogeneity of disease progression. The early involvement of brainstem nuclei such as the DMV may therefore provide a potential anatomical basis for the autonomic dysregulation, including GI dysfunction, that frequently precedes the onset of motor deficits. These interactions occur within the framework of the brain‐gut axis, a complex, bidirectional communication network linking the GI tract and the CNS, enabling the coordinated regulation of digestive function and overall physiological homeostasis. Given its integrative nature, it is not surprising that PD exhibits pathology at multiple levels of this network.

## 3 ENS and PD

The ENS consists of two major neural plexuses that extend throughout the GI tract from the esophagus to the anus. The myenteric plexus, located between the longitudinal and circular muscle layers, controls GI motility by coordinating peristaltic contractions and regulating muscular contractility. In contrast, the submucosal plexuses, located within the connective tissues of the submucosa, regulate epithelial secretion, nutrient absorption, and blood flow to precisely regulate digestive functions and maintain mucosal homeostasis (Sharkey and Mawe 2023). Enteric neurons can be categorized into different functional, morphological, and neurochemical phenotypes: motor neurons, interneurons, intrinsic primary afferent neurons (IPANs), secretomotor, and vasomotor neurons (Furness 2000). Enteric glial cells (EGCs) outnumber enteric neurons in both the myenteric and the submucosal plexuses (Hoff et al. 2008) and comprise irregular, stellate‐shaped cells of multiple distinct types expressing S100, GFAP, SOX8, SOX9, or SOX10 (Hoff et al. 2008; Jessen et al. 1980; Ferri et al. 1982; Gulbransen et al. 2012). While the ENS can independently regulate certain functions, particularly the small and the large intestines, it also receives extrinsic inputs from sympathetic and parasympathetic fibers.

### 3.1 Sympathetic Control of the GI Tract

Briefly, the sympathetic nervous system (SNS) plays a primarily inhibitory role in regulation of GI muscle, mucosal secretion, and blood flow. The preganglionic sympathetic neurons that innervate the GI tract originate from the thoracic and lumbar spinal cord and, as with all other preganglionic autonomic neurons, use acetylcholine as their neurotransmitter. Postganglionic sympathetic neurons, in contrast, use predominantly norepinephrine (NE) as the neurotransmitter; neurons that innervate the stomach are found within the celiac ganglion, whereas neurons innervating the small intestine and colon are located within the superior mesenteric and inferior mesenteric ganglion, respectively (Simmons 1985). The cell bodies of spinal sensory afferents are located in the dorsal root ganglia. They innervate the lower GI tract and can terminate within the muscle layers, as well as in the myenteric and submucosal ganglia, and the mucosa (Chmelir et al. 2025). Sympathetic neurons regulate fluid secretion and absorption through tonic activity on vasoconstrictor and secretomotor neurons (Browning and Travagli 2014). Sympathectomy increases secretion, while stimulation or mimicking sympathetic nerve activity with exogenous NE enhances fluid absorption.

Although sympathetic activity has less direct involvement in the regulation of GI motility (Parsons et al. 1984; Wright et al. 1940; Greenwood et al. 1987), alterations in sympathetic function may contribute to the broader dysautonomia observed in PD. Goldstein and colleagues demonstrated significantly reduced myocardial norepinephrine content in PD patients and further concluded that all PD patients with orthostatic hypotension display some degree of cardiac sympathetic denervation (Goldstein 2007). Furthermore, the decrease in heart rate variability observed under conditions of increased sympathetic activity (e.g., the head‐up tilt table test) suggests impaired sympathetic cardiac modulation (Suzuki et al. 2024). Thermoregulatory dysfunction in PD has likewise been linked to sympathetic impairment; neuropathological studies show α‐syn pathology in hypothalamic nuclei and the intermediolateral cell column, indicating disruption of central thermoregulatory circuitry (Pressnell et al. 2025). Given that norepinephrine release from sympathetic fibers innervating brown adipose tissue regulates non‐shivering thermogenesis, postganglionic noradrenergic loss in thermogenic pathways may contribute to impaired heat production and thermoregulation while impaired sudomotor responses in PD patients also suggest dysfunction within postganglionic sympathetic pathways (Pfeiffer 2012).

### 3.2 Parasympathetic Control of the GI Tract

In contrast, the parasympathetic nervous system (PNS) exerts both an excitatory and inhibitory effects on GI functions. The vagus nerve, the 10th cranial nerve, provides the extrinsic parasympathetic innervation to the stomach, small intestine, and proximal colon. As a mixed sensory: motor nerve, the vagus serves as a bidirectional neural pathway between the brainstem and the GI tract. Preganglionic parasympathetic motoneurons have their cell bodies within the brainstem DMV and innervate postganglionic neurons within myenteric plexus of the GI tract. While all preganglionic DMV neurons are, a priori, cholinergic, postganglionic neurons form one of two pathways: an excitatory cholinergic pathway, activation of which releases acetylcholine to activate muscarinic receptors causing an increase in contractile tone and motility, and a non‐adrenergic, non‐cholinergic (NANC) pathway, activation of which releases predominantly nitric oxide (NO) and/or vasoactive intestinal peptide (VIP) to cause relaxation. Vagal afferents, in contrast, have cell bodies that lie within the paired nodose/jugular ganglia, the central terminals of which enter the brainstem via the tractus solitarius and terminate within the nucleus of the tractus solitarius (NTS). NTS neurons integrate this significant volume of sensory information with inputs from brainstem, midbrain, and cortical nucleus involved in the regulation of autonomic functions, and relay the integrated signal to the adjacent DMV, which contains the vagal motoneurons that provide the motor output to the GI tract via the efferent vagus (Browning and Travagli 2019; Travagli and Anselmi 2016).

Abnormal parasympathetic activity also contributes to the dysautonomia observed in PD. Patients with PD have reduced heart rate variability (HRV), and almost all PD patients with orthostatic hypotension have decreased baroreflex‐cardiovagal responses (Goldstein 2007). PD patients also report bladder overactivity, which presents as nocturnal urinary frequency, sense of urgency, and urge incontinence (Micieli et al. 2003), while Lewy body pathology has been reported in sacral parasympathetic nuclei. Dysregulated parasympathetic regulation of the pancreas has also been reported, with altered pancreatic polypeptide levels observed during hypoglycemic conditions. Indeed, vagal stimulation increases glucagon levels in non‐diabetic hypoglycemic parkinsonian rodents as well as patients (Pham et al. 2025; Taborsky Jr. and Mundinger 2012). Although the focus of this paper is on GI dysfunctions, these findings highlight broader parasympathetic dysregulation of visceral functions in PD.

Collectively, the intrinsic and extrinsic circuitry of the ENS form a tightly regulated system that maintains GI function. Given that GI disturbances are frequently prodromal to motor deficits in the majority of PD patients appearing years, sometimes decades, before the onset of motor symptoms, the ENS emerged as a plausible early target of PD pathology. Growing evidence suggests that α‐syn misfolding occurs within enteric neurons in response to environmental toxins and microbial signals and that their anatomical connectivity with vagal and sympathetic pathways supports the transneuronal spread of the pathology (Pan‐Montojo et al. 2010; Anselmi et al. 2018; Sampson et al. 2016; Holmqvist et al. 2014). In 1984, Qualman and colleagues were the first to report the presence of LB in the myenteric plexus of PD patients (Qualman et al. 1984). Shortly thereafter, another study found LB in the myenteric and submucosal plexuses of all PD patients examined (Wakabayashi et al. 1988). This same study also noted that the LBs were similar to those that are found in the CNS, albeit smaller in size (Pouclet et al. 2012). Strikingly, patients experiencing GI disturbances appear to have an increased risk of developing PD (Poewe 2008). Chronic intestinal inflammatory diseases, including Crohn's disease and ulcerative colitis, are associated with an increased incidence of PD, while long‐term anti‐inflammatory treatment appears to reduce this risk (Villumsen et al. 2019; Camacho‐Soto et al. 2018). Patients diagnosed with disorders of gut‐brain interactions (DGBI) including irritable bowel syndrome have also been found to have an increased likelihood of subsequent PD diagnosis (Liu et al. 2021).

While the presence of (LB) in the ENS indicates α‐syn pathology outside of the CNS, they may not necessarily predict the development of GI symptoms. Increasing evidence suggests, however, that α‐syn‐induced oxidative stress is associated with enteric neurodegeneration thereby contributing to GI dysfunction in PD. Colonic biopsies from diagnosed PD patients not yet receiving dopamine replacement therapy showed an increased intestinal permeability, which correlated with increased staining for *E. coli* bacteria, α‐syn, and 3‐nitrotyrosine, a marker of oxidative stress (Forsyth et al. 2011). Other studies of colonic biopsies provided further support for the role of GI inflammation in PD patients, concluding that pro‐inflammatory cytokine expression is increased in PD and that enteric inflammation is associated with glial activation, as indicated by elevated GFAP expression levels (Devos et al. 2013). Oxidative stress, especially oxidative modifications like nitration, has been shown to induce oligomerization of α‐syn both in vitro and in vivo, the newly formed covalent bonds of which lead to more stable protein inclusions that can withstand denaturation (Giasson et al. 2000; Chavarría and Souza 2013; Uversky et al. 2005; Scudamore and Ciossek 2018). The consistent presence of misfolded α‐syn in human intestinal biopsies suggested its use as a relatively inexpensive biomarker for detecting PD in its earliest stages (i.e., before the development of motor symptoms) (Pouclet et al. 2012; Forsyth et al. 2011; Lebouvier et al. 2010), although it should be noted that standard intestinal biopsies sample only the mucosa (epithelium, lamina propria, and possibly the muscularis mucosa) and biopsies in which neuronal tissue is collected are problematic to obtain. Newer α‐syn seed amplification assays, such as real‐time quaking induced conversion (RT‐QuIC), have been developed, which enable the detection of misfolded α‐syn in intestinal mucosal samples and peripheral biofluids, including blood and serum (Vascellari et al. 2023; Parveen et al. 2025; Okuzumi et al. 2023), which may provide a more readily accessible means of detecting synucleinopathies prior to the development of motor symptoms and disease diagnosis.

Many preclinical animal models of dopaminergic degeneration and parkinsonism support the role of mitochondrial dysfunction and oxidative stress in disease pathology. Both MPTP, a neurotoxin that is metabolized to MPP^+^ and selectively taken up by dopaminergic neurons (He et al. 2024), and rotenone, a lipophilic pesticide that readily crosses the blood–brain barrier (Sherer et al. 2003), inhibit mitochondrial complex I activity, thereby increasing ROS production, and have been used to model PD. In mice, enteric glial cells within the gastric myenteric plexus have been found to have elevated lipid peroxidation, as measured by 4‐HNE levels, after a single dose of MPTP. The same study also found that chronic MPTP exposure led to a prolonged inflammatory state, activation of the NF‐kB pathway, and significantly increased levels of dopamine in the ENS as a possible compensatory mechanism (Heng et al. 2022). The same MPTP mouse model shows a reduction in Nrf2 and antioxidant protein expression in gastric and colonic samples, suggesting a decreased ability for enteric neurons to counteract oxidative stress (Sampath et al. 2019).

Similar pathological features have been observed in rotenone‐based models, where chronic administration induces glial activation and oxidative stress (Murakami et al. 2015). A similar finding regarding the importance of oxidative stress in the gut and parkinsonian pathology came from another study using the rotenone model, where the activity of asparagine endopeptidase (AEP), known for cleaving human α‐syn and promoting its aggregation, is increased by ENS oxidative stress. In contrast, knocking out AEP reduces motor deficits, prevents the spread of LBs from the gut to the brain, and diminishes neuroinflammation. (Wang et al. 2023).

Although the presence of oxidative stress and glial activation in the ENS is well established, whether PD or parkinsonism induces enteric neurodegeneration is still open to debate. Since the hallmark of PD is the loss of dopaminergic neurons in the SNpc, studies have examined colonic tissue from PD patients to determine if the same principle applies to the ENS. While differences in neuronal numbers were not detected, reduced enteric dopaminergic levels in PD patients with chronic constipation (Singaram et al. 1995) were identified, as well as a significant decrease in VIP levels in the submucosal plexus of patients with PD and chronic constipation compared to those with chronic constipation alone, supporting a role for downregulation of VIP expression rather than neuronal loss (Giancola et al. 2017). Results from animal models, however, are contradictory; some studies have observed a loss of dopaminergic ENS neurons accompanied by increased colonic contraction following 10 days of MPTP administration (Anderson et al. 2007). Of note, however, although these findings are described as resulting from dopaminergic neuron death, immunohistochemical assessment relied on tyrosine hydroxylase (TH) staining, which could also be indicative of reduced TH expression rather than cellular death per se. A 6‐ODHA, microinjection lesion model targeting the SNpc, study concluded that nitrergic myenteric neurons increase in number without reduction in the overall neural count (Toti and Travagli 2014). In contrast, long‐term subcutaneous rotenone administration via an osmotic pump caused significant neurodegeneration in the myenteric plexus (Murakami et al. 2015). Collectively, these data suggest that enteric oxidative stress, inflammation, and neurodegeneration may contribute to prodromal Gi dysfunctions, including delayed gastric emptying and constipation, which are commonly observed in PD patients.

Thus, while GI disturbances may be caused by enteric neuron dysregulation or neuronal loss, it may also result from CNS dysregulation and disruption of the brain‐gut axis, either at the level of the brainstem DMV or at the level of the SNpc, which has been shown to tonically regulate DMV activity and vagal efferent control of the GI tract (Anselmi et al. 2017). Consistent with this, in multiple experimental models, including MPTP (Anderson et al. 2007), rotenone (Drolet et al. 2009), and 6‐OHDA (Toti and Travagli 2014), GI dysfunction has been observed following central dopaminergic degeneration. Regardless of whether GI α‐syn aggregates are the primary or secondary events in the observed GI dysfunction in PD, these studies in the ENS are essential to understanding disease pathology and the role of the gut‐brain (or brain‐gut) axis.

## 4 Brainstem and PD

The brainstem, especially the DMV and the locus coeruleus (LC), has been suggested to be the first region in the CNS to display α‐syn pathology, with Braak and colleagues proposing that misfolded α‐syn is transported from the ENS to the DMV via the efferent vagus nerve. Supporting this, retrospective analyses of patients who received prior truncal vagotomy showed a reduced incidence of PD. Although reanalysis of the same data raised counterarguments, such as confounding surgical factors and variability across cohorts (Svensson et al. 2015), many preclinical experiments confirmed that the vagus nerve is involved in the retrograde transport of α‐syn. The first evidence came from findings showing that injecting human PD brain lysate containing α‐syn into the intestinal wall resulted in timely transport of α‐syn to the DMV via the vagus nerve (Holmqvist et al. 2014), a phenomenon since replicated across multiple animal models. Injection of preformed α‐syn fibrils into the duodenal and pyloric muscle layers, for example, led to the detection of α‐syn in the DMV, LC, and SNpc, which was prevented by truncal vagotomy (Kim et al. 2019; Uemura et al. 2018). Similarly, a subdiaphragmatic vagotomy prevented the ascending spread of α‐syn pathology and subsequent parkinsonism in an environmental rodent model (Anselmi et al. 2018).

The intrinsic membrane properties of DMV neurons endow them with tonic pacemaking activity, but the excitability and activity of DMV neurons are regulated by synaptic inputs, particularly tonic GABAergic inhibition from the adjacent NTS. DMV neurons also receive glutamatergic and catecholaminergic signals from the NTS, however, these do not appear to regulate the tonic activity of vagal efferents under basal conditions. Importantly, the DMV receives direct dopaminergic projections from the SNpc—the monosynaptic nigro‐vagal pathway—which appears to modulate parasympathetic output to the GI tract. Not only does activation of the SNpc via microinjection of the glutamate agonist, NMDA, increase gastric and proximal colon motility, but optogenetic inhibition of this nigro‐vagal pathway decreases gastric tone and motility in a manner dependent upon the efferent vagus activation of brainstem dopamine D1 receptors (Anselmi et al. 2017). This nigro‐vagal pathway may be the essential anatomical link between ingestion of environmental toxins, induced misfolding of α‐syn, gut‐brain axis dysfunction and central dopaminergic neurodegeneration responsible for the development of PD.

Similar to the ENS, neuronal dysregulation, oxidative stress, and glial activation have been observed in the brainstem in preclinical parkinsonian models. In an environmental model of rodent parkinsonism, for example, excitatory transmission to DMV neurons was increased, possibly as a form of maladaptive plasticity to compensate for observed GI dysfunction, including gastric hypomotility and delayed gastric emptying (Bove et al. 2019). Similarly, in a mutant α‐syn overexpressing mouse parkinsonian model, α‐syn accumulation induces potassium Kv4 channelopathy in vagal motoneurons, leading to impaired excitability of DMV neurons and reduction of gastrointestinal motility (Chiu et al. 2021). In an attempt to investigate the mechanistic basis of extranigral susceptibility in parkinsonism, Musgrove et al. showed that, following injection of high volumes of AAV‐mediated human α‐syn directly into the vagus nerve, the resulting α‐syn misfolding leads to increased oxidative stress and loss of DMV cholinergic neurons. This same study additionally demonstrated that inhibiting microglial NADPH‐oxidase reduced oxidative stress and prevented DMV neurodegeneration (Musgrove et al. 2019). Finally, because DMV neurons exhibit calcium‐dependent autonomous pacemaking, they have been shown to exhibit elevated calcium influx and weak buffering capacity, leading to mitochondrial oxidative stress, which is believed to contribute to their vulnerability and the development of early dysautonomia in PD (Cooper et al. 2015; Goldberg et al. 2012).

Nevertheless, despite their critical role as potentially the first neurons affected by prodromal parkinsonian pathology and the site of first entry of misfolded α‐syn into the CNS, the role of DMV neurons in PD pathogenesis remains understudied. The involvement of oxidative stress in DMV neurons, in particular, deserves further study given the potential of brainstem vagal neurons as an early pathological site of central involvement. Together, these findings support the role of the brainstem, not only as a conduit of brain‐gut (or gut‐brain) information relay, but is also an early site of α‐syn pathology and cellular alterations in PD. The selective vulnerability of SNpc neurons to degeneration suggests, however, that additional intrinsic properties contribute to disease progression. Elucidating the intrinsic features that predispose these neurons to oxidative stress helps connect autonomic dysfunction and neurodegeneration in PD.

## 5 SNpc and PD

The SNpc contains primarily dopaminergic neurons that form the nigrostriatal pathway, which is critical for modulating voluntary movement through their projections to the dorsal striatum, and the degeneration of this pathway leads to cardinal motor symptoms of PD. Several intrinsic properties render SNpc dopaminergic neurons particularly vulnerable to oxidative stress. These neurons are highly arborized projection neurons with physiological properties that impose unusually high metabolic demand, as the ATP required to restore membrane potential after action potential firing is directly proportional to the size and arborization of the neuron (Pissadaki and Bolam 2013). Their L‐type calcium channel‐mediated autonomous pacemaking activity further exacerbates this intrinsic vulnerability, which requires nuanced calcium buffering, which is another contributor to the increased cellular metabolic load (Guzman et al. 2009). They are also the recipients of dense glutamatergic synaptic inputs, particularly from the subthalamic nucleus, which can lead to oxidative stress and excitotoxicity (Rodriguez et al. 1998). SNpc neurons are also characterized by the accumulation of neuromelanin, a byproduct of cytosolic dopamine oxidation; while neuromelanin sequesters toxic materials, it can be released when a neuron degenerates, thereby exacerbating oxidative stress (Zucca et al. 2014).

One of the earliest pieces of evidence for the critical role of oxidative stress came from analysis of levels of glutathione (GSH), a major intracellular antioxidant, in postmortem human PD brain samples. Studies reported a significant reduction in GSH levels in the SNpc and, further, that this reduction was specifically localized to the nigral region (Perry et al. 1982). Around the same time, although the action of mechanism was identified later, a breakthrough in support of the oxidative stress hypothesis of PD came from the observation that the intravenous administration of MPTP, a street‐drug manufactured as a substitute for heroin, among multiple young adults led to the development of parkinsonism which was recovered by administration of L‐DOPA and this phenomenon was recapitulated in non‐human primates and rodents (Langston et al. 1999; Burns et al. 1983; Davis et al. 1979; Fredriksson et al. 1990). Dexter et al. subsequently demonstrated that basal levels of malondialdehyde, an intermediate in the lipid peroxidation process, are elevated in the SNpc of PD brains, providing evidence for oxidative damage, particularly to the neuronal membranes (Dexter et al. 1986). Similarly, Saggu et al. showed that the particulate form of superoxide dismutase, the enzyme responsible for antioxidant activity, is reduced in the SNpc and interpreted this as a possible compensation for elevated levels of superoxide (Saggu et al. 1989).

Glial activation within the SNpc is a well‐recognized feature of PD. The first major evidence for the role of microglia and macrophages in nigral dopaminergic degeneration was provided by McGeer et al., who detected elevated levels of human leukocyte antigen (HLA‐DR) in the SNpc of PD patients (McGeer et al. 1988). Subsequent work in postmortem neuropathological analysis of brains from individuals who developed parkinsonism due to MPTP exposure also revealed microglial activation and was associated with the accumulation of extraneuronal neuromelanin (Davis et al. 1979). The ongoing and persistent activation of microglia, even years after MPTP exposure, indicates a continuous cycle of inflammation which may be involved in continued cellular death (Langston et al. 1999). Since PD patients are almost always treated with L‐DOPA, it was necessary to further test whether activated microglia result from disease pathology rather than treatment. A study on MPTP‐injected non‐human primates further validated the presence of microglial activation a year after the last MPTP injection and concluded that microglial activation is due to toxin exposure rather than L‐DOPA (Burns et al. 1983). In the search for a mechanism, Wilms et al. exposed microglial cultures to neuromelanin, which led to the upregulation of tumor necrosis factor alpha, interleukin‐6, and nitric oxide subsequent to impairment of the proinflammatory transcription factor nuclear factor (NF‐ΚB) via phosphorylation by IΚB kinase (Wilms et al. 2003). In contrast, inhibition of IΚB decreased the release of neurotoxic mediators. These findings suggest a possible role of neuromelanin in PD pathogenesis, particularly to the chronification of inflammation.

More recent work has demonstrated that, not only is microglial activation in PD persistent, but it is also heterogeneous and highly dynamic, characterized by a spectrum of neurotoxic and neuroprotective phenotypes. In vivo PET imaging confirmed microglial activation in PD patients, whereas single‐cell sequencing revealed differential gene expression and altered signaling pathways suggestive of a pro‐inflammatory activation trajectory with key transcription factors such as Nfe2l2 and Runx1 mediating microglial activation in a MPTP‐induced PD mouse model (Liu et al. 2022, 2024). Furthermore, in the α‐syn preformed fibril (PFF) model of PD, microglial activation via LPS‐induced inflammation actively propagates α‐syn and exacerbates neuronal injury, whereas inhibiting microglial activation with colony‐stimulating factor 1 receptor (CSF1R) inhibitors significantly reduces α‐syn accumulation, dopaminergic neuron loss in the SNpc, and improves motor function (Lai et al. 2024).

Due to the bidirectional interaction between microglia and astrocytes, understanding astrocyte function is also crucial to elucidating PD pathology. Similar to microglia, astrocytes have a dual role in PD, both secreting neuroprotective factors such as glial cell line derived neurotrophic factor (GDNF) and mesencephalic astrocyte‐derived neurotrophic factor (MANF) as well as releasing pro‐inflammatory cytokines such as TNF‐a and IL‐1B, in addition to ROS production, all of which may worsen neuronal injury (Rappold and Tieu 2010). Wakabayashi et al. were among the first to report astrocytic alterations in the midbrain of PD patients, indicating GFAP immunoreactivity and the presence of Gallyas‐positive, tau‐negative cytoplasmic inclusion in glial cells (Wakabayashi and Takahashi 1996). Functional studies revealed conflicting results about the role of astrocytes in PD pathology, however. Immunohistochemical assessment of SNpc astrocyte morphology in postmortem PD patient samples showed retracted astrocyte processes and amoeba‐like microglia near dying or dead neurons, whereas SNpc neurons from healthy controls are tightly wrapped with astrocyte processes (Knott et al. 1999). Supporting this, GFAP levels in the SNpc have been inversely correlated with α‐syn accumulation (Tong et al. 2015). Conversely, selective expression of mutant α‐syn in astrocytes led to motor deficits, loss of dopaminergic and motor neurons (including dopaminergic neurons in the SNpc and motor neurons in the cervical and lumbar spinal cord), and downregulation of astrocytic glutamate transporters, suggesting compromised astrocyte function (Gu et al. 2010).

Accumulation of misfolded α‐syn in astrocytes can trigger a defense mechanism in neighboring health astrocytes through the formation of tunneling nanotubules (TNTs), actin‐based membrane bridges that allow for the exchange of cellular content and signaling molecules between neurons, astrocytes, and microglia (Rustom et al. 2004; Gurke et al. 2008). Engulfment of misfolded α‐syn In human embryonic stem cell‐derived astrocytes triggers mitochondrial fragmentation, swelling of the endoplasmic reticulum, dysfunctional autophagy‐lysosome complexes, and the formation of TNTs, which enable the F‐actin depolymerization‐dependent (i.e., cytochalasin B sensitive) exchange of mitochondria for transport of α‐synuclein to healthy astrocytes (Rostami et al. 2017). Conversely, however, TNTs have also been implicated in the transport of prions; in the context of the body first PD perspective, this may explain the retrograde prion‐like transportation of α‐syn from ENS neurons to the peripheral terminals of vagal efferents and soma brainstem DMV neurons, and then to other CNS regions (Gousset et al. 2009). Indeed, neurons have been shown to transfer α‐syn to astrocytes in an endocytosis‐dependent manner in both in vivo and in vitro PD models. The accumulation of α‐syn within astrocytes alters their gene expression profiles to favor a pro‐inflammatory response, which may contribute to and sustain disease pathology and may be an initial step towards the observed neurotoxic astroglial response (Lee et al. 2010).

Given their role as major ROS producers, SNpc mitochondrial dysfunction has attracted substantial attention as a driver of PD etiology. Early studies identified a notable reduction in mitochondrial complex I activity in the SNpc of patients with PD, without a compensatory increase in mitochondrial mass (Schapira et al. 1990). Genetic mutations associated with PD, including PINK1, Parkin, and DJ‐1, further implicate dysregulated mitochondrial function and weakened antioxidant defenses in disease pathophysiology (Cookson 2004). Mitochondrial dysfunction leads to the release of damage‐associated molecular patterns (DAMPs), such as mitochondrial DNA (mtDNA) fragments, which activate microglial innate immune responses via Toll‐like receptor 9 (TLR9), NLRP3 inflammasome, and stimulator of interferon genes (STING)‐dependent pathways (Maatouk et al. 2018; Sarkar et al. 2017; Hinkle et al. 2022). Microglial activation increases neuroinflammation and exacerbates mitochondrial damage, creating a vicious cycle that promotes neurodegeneration. Mitochondrial dysfunction also promotes apoptosis of dopaminergic neurons through the release of cytochrome c into the cytoplasm, which triggers the activation of caspases 3 and 9 leading to programmed cell death. Mitochondria‐mediated apoptosis aligns with the gradual and subtle progression of nigral degeneration observed in PD.

Dopamine can itself also contribute to increased oxidative stress levels by generating reactive species such as hydrogen peroxide, superoxide radicals, hydroxyl radicals, and dopamine‐quinones through autoxidation following cytosolic interaction with oxygen (Heikkila and Cohen 1973). Under physiological conditions, the acidic environment of synaptic vesicles stabilizes dopamine and protects it from autoxidation (Umek et al. 2018). In PD, however, elevated dopamine levels in the cytosol, where the pH is neutral and oxygen is present, lead to dopamine autoxidation, increased α‐syn aggregation and impaired vesicular storage (Lee et al. 2011; Mosharov et al. 2009). Furthermore, in cultured midbrain neurons, L‐DOPA‐induced elevation of cytosolic dopamine levels and mitochondrial oxidative stress are significantly higher in SNpc DA neurons than in VTA DA neurons, likely due to cytosolic Ca^2+^ entry through L‐type calcium channels. Indeed, both pharmacological blockade of L‐type calcium channels with dihydropyridine and genetic knockout of α‐syn reduce cytosolic dopamine and protect SNpc neurons from L‐DOPA‐induced cell death (Mosharov et al. 2009). In support of this, vesicular monoamine transporter 2 (VMAT2) levels oppose dopaminergic toxicity in the MPTP mouse model, suggesting that VMAT2‐mediated vesicular storage may offer protection against cytosolic dopamine‐induced cellular degeneration (Lohr et al. 2016). The complex interaction between Ca^2+^, cytosolic dopamine, and α‐syn may help to explain the vulnerability of SNpc neurons in PD.

## 6 Microbiome and PD

The gut microbiome is increasingly recognized as a key modulator of the brain‐gut axis in PD. Gut microbial and their metabolites regulate epithelial barrier integrity and mucosal immune activation. Clinical studies consistently report alterations in gut microbiota compositions and microbial metabolites in PD patients (Nishiwaki et al. 2020), including reduced levels of short‐chain fatty acids (SCFAs) and changes in immune signaling, even in early or treatment naïve stages of the disease (Bedarf et al. 2017; Unger et al. 2016; Hasegawa et al. 2015). Preclinical studies further support the role of microbiota in PD pathogenesis. Colonizing α‐syn overexpressing mice with microbiota from PD patients led to the development of motor deficits, increased activation of microglia and α‐syn pathology in the SNpc, while the bacterial depletion attenuated the disease severity (Sampson et al. 2016). Although these studies do not indicate a causal relationship between dysbiosis and the development of PD, they do suggest that microbiome‐associated changes can modulate neuroinflammation and disease phenotype in vivo, supporting microbiome‐targeted interventions as a potential disease‐modifying therapeutic strategy. In particular, alterations in microbial composition and metabolites may increase epithelial barrier permeability, local inflammation, mitochondrial dysfunction and oxidative stress, all of which have been implicated in α‐syn misfolding (Forsyth et al. 2011; Abdel‐Haq et al. 2022; Morais et al. 2025). Indeed, prebiotic supplementation has been shown to alter microbiome composition, increase SCFA‐producing bacteria, and improve GI symptoms in PD patients (Hall et al. 2022) as well as in α‐syn overexpressing mice (Abdel‐Haq et al. 2022).

## 7 Conclusion

PD is increasingly recognized as a multisystem disorder in which extranigral pathological processes contribute to disease progression. Evidence from clinical and preclinical studies supports the existence of both brain‐first and body‐first trajectories of disease etiology, which may contribute to the heterogeneity of the patient population. The body‐first subtype of PD emphasizes the involvement of the GI tract, DMV, and SNpc, each displaying pathological features such as oxidative stress, mitochondrial dysfunction, glial activation, and α‐syn misfolding, which may contribute to their vulnerability and the development of non‐motor symptoms. At the same time, studies also demonstrate that dysregulation of the GI tract may arise from degeneration of dopaminergic neurons in the SNpc, highlighting the bidirectional nature of the brain‐gut axis in PD. Better understanding of how pathological processes along the brain‐gut axis will be essential to targeting of specific therapeutic intervention strategies towards the prodromal non‐motor symptoms and may help identify biomarkers for early detection prior to disease diagnosis. Early intervention targeting oxidative stress and glial activation, for example, may enable a shift from symptomatic dopamine replacement towards disease‐modifying therapeutic strategies.

## Funding

The authors wish to thank NIH grants NIDDK 124098 and DoD Grant W81XWH2110915 for their support.

## Conflicts of Interest

The authors declare no conflicts of interest.

## Data Availability

The authors have nothing to report.
